# Presence of Human Herpes Virus 6 (HHV6) in pediatric lymphomas: impact on clinical course and association with cytomegalovirus infection

**DOI:** 10.1186/1743-422X-7-287

**Published:** 2010-10-27

**Authors:** Samah A Loutfy, Mohamed Fawzy, Mohamed El-Wakil, Manar M Moneer

**Affiliations:** 1 Virology and Immunology Unit, Cancer Biology Department, National Cancer Institute, Cairo University, Egypt; 2 Pediatric Oncology Department, National Cancer Institute, Cairo University, Egypt; 3 Clinical Oncology Department, Faculty of Medicine, Beni-Suef University, Egypt; 4 Epidemiology & Biostatistics Department, National Cancer Institute, Cairo University, Egypt

## Abstract

**Background:**

Activation of herpes virus 6 (HHV6) has seen in Hodgkin's and non-Hodgkin's Lymphoma (HL&NHL) as a result of lymphoma associated immunosuppression. Multiple studies have suggested an association between both HHV6 and cytomegalovirus CMV for development of CMV disease affecting the pathogenesis of lymphoma. Therefore, this study investigated the frequency of HHV6, its impact on clinical manifestations of lymphoma and its possible association with risk for development of CMV infection in pediatric lymphoma patients.

**Methods:**

Presence of HHV6 DNA and CMV DNA was investigated by PCR assay in both WBC's and plasma samples from 50 patients diagnosed with HL or NHL. CMV antibody titer was also determined in sera obtained from each patient. Twenty apparently healthy siblings were used as a control group.

**Results:**

In a study group of 50 patients diagnosed with HL or NHL, 23/50 (46%) were found to be positive for herpes virus DNA (HHV6 or CMV) in WBC's or plasma by PCR assay and this was significantly higher than its presence in the pediatric control group 2/20 (10%) (p = 0.005). Ten out of these 23 (43%) were found to have active CMV infection. Fifty six percent of patients with CMV infection were found among NHL cases with B- subtype. The presence of both herpes viruses DNA was significantly associated with more frequent episodes of febrile neutropenia (median 3 episodes), absolute neutrophil count (< 0.8), lymphocytes (< 0.5), and low hemoglobin level (< 9.1), (p < 0.05).

**Conclusion:**

The presence of HHV6 can be considered as a predicting indicator of cellular immunosuppression preceding the onset of CMV infection which may result in a severe outcome among pediatric lymphoma patients.

## Introduction

Human herpesvirus 6 (HHV6) was first reported in 1986, as human B-lymphotropic virus. Name was subsequently changed to human herpesvirus 6 as its tropism was further characterized [1,2] and it was identified as a member of the β family of herpes viruses [3]. Seroepidemiological surveys have shown that HHV6 is highly prevalent in human populations in different geographical areas with prevalence varying between 70 and 100% [4]. HHV6 shares with other members of the human Herpesviridae family an ability to cause latent infection with reactivation during periods of immunosuppression [5]. Also, HHV6 and CMV share a tropism for cells of the immune system [6] and for induction of immunosuppression [7]. These similarities, together with the ability of HHV-6 to reactivate heterologous virus [8], may explain its role in the pathogenesis of CMV disease in an immunocompromised host, such as post transplant patients, with respect to CMV disease and the development of opportunistic fungal infections [8].

Pediatric clinical presentations of HHV-6 infection vary depending upon the age and immune competence of the child. In the immunocompromised host, the spectrum of specific HHV6 clinical syndromes remains undefined [9] but is associated with a worse outcome [10]. HHV6 reactivation occurs in 33-48% of patients undergoing hematopoietic stem cell transplantation and is associated with organ-specific diseases such as pneumonitis, hepatitis, encephalitis, bone marrow suppression and non specific febrile syndromes [10].

Activation of HHV6 was seen in both HL and NHL as a result of lymphoma associated immunosuppression and variation in its frequency was reported [11]. In National Cancer Institute of Egypt, there are very limited reports about the role of HHV6 infection in pediatric lymphomas and its association with CMV activation.

For this reason the focus of this study was, i) to investigate the presence of HHV6 in white blood cells and plasma of the children with lymphoma, ii) to study the impact of HHV6 on the clinical features of pediatric lymphoma disease, iii) Investigate frequency of CMV infection and its impact upon the course of the disease.

## Patients and Methods

### Patients

This cross sectional study was conducted on 50 pediatric lymphoma patients (Hodgkin's & Non Hodgkin's) diagnosed and treated at the Pediatric Oncology Department, National Cancer Institute (NCI), Cairo University between September 2007 and October 2008. Twenty patients individuals' were included as matched controls. The Institutional Review board (IRB) of the NCI approved the protocol. Informed written consent was obtained from guardians of all children enrolled in the study.

The study included patients between 1 and 16 years old of both sexes. Then, all patients were thoroughly evaluated for clinicopathological data.

Disease extent and staging were established after a detailed history and physical assessment. Full local and systemic imaging surveys (X-rays, CTs, MRI) according to disease site and clinical presentation were performed. Gallium scan and bone marrow aspirate (±) trephine biopsies were also performed when indicated by treatment protocol. Other baseline and prognostic investigations were carried out; serum Lactate dehydrogenase (LDH), Erythrocyte sedimentation rate (ESR), Complete blood count (CBC), liver and renal function tests.

The Ann Arbor staging system [12] was used for Hodgkin's lymphoma (HL), where patients received tailored courses of cychemotherapy in form of "ABVD" (±) involved field radiation of reduced dose (25 GY) according to their risk stratification [13].

Non Hodgkin lymphoma patients were staged according to St Jude Children Staging System [14]. B cell lymphomas were risk stratified and treated according to the SFOP (French Society of Pediatric Oncology) protocols [15]. The BFM protocol [16] was used for T cell precursor lymphoblastic lymphoma.

Patients under study have been interviewed at different phases of therapy and medical records were reviewed for clinical progress evaluation and data abstraction.

### Histopathology and immunohistochemistry

All 50 tumor tissue specimens were pathologically restudied on the basis of the examination of hematoxylin-eosin and Giemsa-stained slides. Tumor type was specified for each case according to the current World Health Organization classification [17]. All cases were further stained for basic B and T cell markers, and immunohistochemistry for NHL sub-classification. All cases of HL were stained for CD15 and CD30 and staining for CD20 and CD45RA was performed if NLPHL (Nodular lymphocyte-predominant Hodgkin's lymphoma) was suspected

## Specimen collection

Blood specimens were collected into tubes with EDTA (Ethylenediaminetetraacetic acid) anticoagulant, left at room temperature for 1 hour to sediment erythrocyte. The plasma was separated and centrifuged at 800 × g for 10 min. Samples of plasma were stored at -20°C for DNA extraction and detection of HCMV IgG by ELISA. Leukocytes were isolated according to the protocol of Vander Bij et al [18] and stored in aliquots of 100 μl saline at -40°C until nucleic acid extraction.

## Molecular detection

### 1 - Nucleic acid extraction

Viral DNA was extracted from both WBCs and plasma specimens using GFX Genomic Blood DNA Purification Kit (Amersham Biosciences, UK) according to the manufacturer's instructions. DNA extracts were placed on ice and used immediately for PCR or stored at -40°C until analysis. The amount of viral DNA was measured by spectrophtometry using a Nano-Drop 2000 spectrophotometer (Thermo Scientific/US, Canada) and 100 ng of DNA template was used in the PCR assays.

A sample for each participant in the study was subjected to one round of PCR for detection of HHV-6 DNA and nested PCR for detection of HCMV (human cytomegalovirus) in both cells and plasma specimens. For HHV6 positive and negative (water) controls were run in each PCR assay. For HCMV, AD169 DNA from CMV reference strain AD169 (American Type Culture Collection, Rockville, Md., USA) was extracted by the same extraction procedure and used as a positive control in the PCR assay.

### 2 - Amplification of HHV-6 DNA

HHV6 DNA was selectively amplified by PCR using sequence-specific primers for a 249-bp portion of the large-tegument protein gene of HHV-6 between nucleotides 27240 and 27483 by following the method of Osiowy et al [19]. This primer set has previously been shown to be specific for HHV-6 and not to amplify DNA from other members of the herpesvirus family including (CMV), Epstein-Barr virus, varicella zoster virus and herpes simplex viruses 1 and 2. PCRs were performed in a final volume of 20 μl, which included 5 μl of DNA extract, 1 U of Taq DNA polymerase (Boehringer Mannheim, Canada), 0.25 μM (each) primer, 200 μM dNTPs, 10 mM Tris HCl (pH 8.3), 1.5 mM MgCl_2 _and 50 mM KCl. The samples were overlaid with mineral oil to prevent evaporation.

Thermocycling conditions were as follows: (i) an initial denaturation step of 2.5 min at 94°C; (ii) 35 cycles, with 1 cycle consisting of 30 s of denaturation at 94°C, 30 s of annealing at 62°C, and 50 s of extension at 72°C; and (iii) a final extension step of 5 min at 72°C. PCR was performed in a Perkin Elmer-Cetus thermocycler.

### 3 - Amplification of HCMV

HCMV-DNA was detected using sequence-specific primers for a 147-bp portion of the CMV immediate early gene between nucleotides 172750 and 172895 by the method of Vogelberg [20]. PCR was performed using a ready made GEN-Master Mix (BIORON, Germany). Five μl of DNA extract was included in the reaction mixture containing 10× Tris HCL buffer, dNTPs (0.4 mM), MgCl_2 _(1.5 mM), 0.02% Tween-20, 0.1 units/μl DFS-Taq DNA polymerase (BIORON, Germany), along with 0.6 μM of each primer, in a final volume of 25 μl. The samples were overlaid with mineral oil to prevent evaporation. The reaction was performed in a DNA thermal cycler (Perkin Elmer-Cetus): (i) an initial denaturation step of 9 min at 95°C; (ii) 30 cycles, with 1 min cycle consisting of 1 min of denaturation at 95°C, 1 min of annealing at 55°C, and 1 min of extension at 72°C; and (iii) a final extension step of 7 min at 60°C. Using 10 fold dilutions of the viral DNA, the sensitivity of the PCR assay for CMV was determined according to Brown [21]. The target CMV DNA could be amplified and detected with sensitivity of 10 copies of viral genome/ug genomic DNA [22].

### 4 - Detection of Amplified products by agarose gel electrophoresis

**Figure 1:**
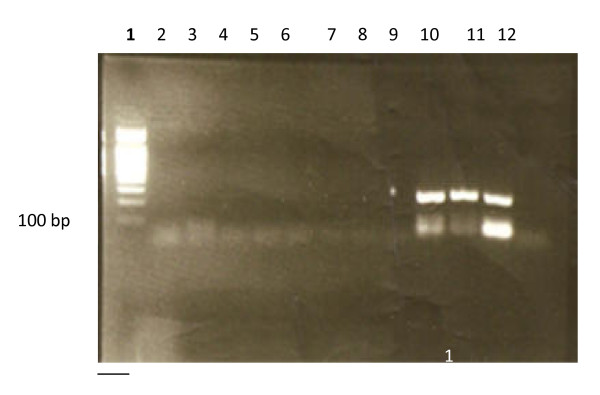
**EB-stained gel electrophoresis of HHV-6-DNA PCR product showing positive lanes (10-12) and negative lanes (2-9)**. 100 bp ladder lane (1). N.B. positive signals are 249 bp., Lane 2 is negative control (water). Lane 12 is positive control

15 μl of PCR product was subjected to electrophoresis on a 2.5% agarose gel (Sigma) in Tris-Acetate buffer (TAE 1X) pH 8.2, stained with 0.5 ug/ml ethidium bromide and examined under UV transillumination and photographed. Product sizes were estimated by comparison with 100 bp DNA ladder (Amersham, UK). Amplified fragments are 249 bp for HHV-6 (Figure 1) and 147 for HCMV [22].

## Serological detection of HCMV IgG by ELISA

Analysis of serum samples for IgG antibodies to CMV was performed using CMV biokit, S.A.08186 (Barcelona-Spain) or bioelisa CMV IgG ELISA test according to manufacturer's instructions kit. The concentration of antibodies in the sample was estimated using a calibration curve. Antibody concentration > 0.25 IU/ml was taken to indicate immune status. A level > 2.5 was considered a high positive and implies he had recent or reactivated CMV infection [23].

### Statistical methods

Data were statistically described in terms of median and range or mean and standard deviation (± SD), frequencies and percentages, as appropriate. Comparison of quantitative variables was done using Mann Whitney *U *test for independent samples in 2 groups and Kruskal Wallis test in comparing more than 2 groups. For comparing categorical data, Chi square (Fisher's exact) test was performed. A *p *value < 0.05 was considered statistically significant. All statistical calculations were done using SPSS version 15 (SPSS Inc., Chicago, IL, USA).

## Results

The median age of the 50 pediatric lymphoma patients was 8.5 years (range 2-18 yrs), and that of apparently healthy siblings was 7 years (range 1-18 yrs). Preponderance of males was observed among lymphoma patients with a male to female ratio of 1.7:1, and 1.5:1 for normal siblings. There was no significant difference between lymphoma and normal siblings regarding age and sex (p = 0.128 and 0.754, respectively). Clinical parameters for lymphoma patients are summarized in Table 1. Lymphoma group included 17 patients with Non-Hodgkin's lymphoma (NHL) and 33 with Hodgkin's disease (HL); their subtypes are shown in Table 2. Half of the lymphomas cases were at an early stage of disease (stage 1 and 2) and the other half were at later stages (3, 4). Eleven patients with stage 1 and 2 disease received radiotherapy. Radiotherapy was also given to three patients with stage 3 and 4 disease. Twenty four patients were in induction phase of therapy, 23 were at maintenance phase of therapy, and 3 received salvage treatment for relapsing disease.

**Table 1:** Clinical and Laboratory characteristics of pediatric lymphoma patients

Variables	HL N = 33	NHL N = 17
**Age ***(Mean ± SD)*	10.5 ± 4.5	8.1 ± 4.6
**Sex**		
Male: n = 32	18	14
Female: n = 18	15	3
M:F ratio	1:2:1	4:6:1
**LFT ***(Mean ± SD)*		
ALT (IU/L)	25.2 ± 11.7	29.2 ± 12.7
AST (IU/L)	21.2 ± 11.4	21.7 ± 9.9
T. Bil (mg/dL)	1.1 ± 0.3	1.0 ± 0.4
**KFT ***(Mean ± SD)*		
Creatinine (mg/dL)	0.9 ± 0.3	1.0 ± 0.3
**CBC ***(Mean ± SD)*		
Hb (g/dL)	10.6 ± 2.2	7.6 ± 3.0
TLC (×109/l)	5.3 ± 3.6	2.9 ± 4.6
Plt (×109/l)	134.9 ± 79.1	171.3 ± 208.4
ANC (×109/l)	1.8 ± 1.3	1.4 ± 2.3
Mono (×109/l)	1.5 ± 1.2	0.6 ± 1.0
		
**LDH ***(Mean ± SD) *(IU/L)	207.4 ± 214.2	443.7 ± 667.5
**ESR 1^st ^hr ***(Mean ± SD)*	40.5 ± 31.1	35.4 ± 24.5
**ESR 2^nd ^hr ***(Mean ± SD)*	73.5 ± 63.7	65.1 ± 45.9
**Phase of therapy**		
Induction: n = 24	15	9
Post-induction: n = 23	16	7
Salvage: n = 3	2	1
**Stage of disease**		
I+II (n = 25)	19	6
III+IV (n = 25)	14	11

SD: Standard deviation, LFT: Liver function test, ALT: Alanin aminotransferase, AST: Aspartate aminotransferase, T. Bil: Total bilirubin, KFT: Kidney function test, CBC: Complete blood picture, Hb: Hemoglobin concentration, TLC: Total leukocytic count, Plt: Platelet count, ANC: Absolute neutrophilic count, Mono: monocyte, LDH: Lactate dehydrogenase, ESR1: Erythrocyte sedimentation rate 1^st ^hour, ESR2: Erythrocyte sedimentation rate 2^nd ^hour

**Table 2:** Lymphoma cases associated with HHV6 infection and both herpes viruses

Lymphoma subtype	No of cases n(%)	HHV6 n = 6(%)	Both n = 10(%)
**NHL (n = 17)**			
B cell lymphoma	16(94)	1(6)	8(47)
Burkitt lymphoma	7(45)	1(6)	4(25)
Burkitt like	5(30)	0(0)	1(6)
Large B cell lymphoma	4(25)	0(0)	3(19)
T-Lymph Lymph	1(6)	0(0)	0(0)

**HL (n = 33)**			
Mc	13(39)	2(6)	2(6)
Lp	8(24)	1(3)	0(0)
Ns	8(24)	1(3)	0(0)
Ld	4(12)	1(3)	0(0)

NHL: Non-Hodgkin's lymphoma, HL: Hodgkin's lymphoma, Mc: multicellularity, Lp: lymphocytic predominance, Ns: nodular sclerosis, Ld: lymphocyte depletion, HHV6: herpes virus 6 single infection, Both: positive for HHV6/CMV DNA.

According to the PCR results in cells or plasma, participants fall in one of 4 categories; those positive for H6 alone, positive for CMV alone, patients positive for both HHV6 and CMV and those negative for both viruses.

### The presence of herpes viruses (HHV6 and/or CMV) in lymphoma patients

Herpes viruses (HHV6 and/or CMV) were present in 23 of the 50 in lymphoma patients (46%), a frequency that was significantly higher than that in the control group (2/20, 10%) (p = 0.005). There was no significant difference between NHL and HL groups regarding presence of HHV6 and/or CMV (10/17, 59% vs. 13/33, 39%, respectively, p = 0.192). However, as shown in table 3, there was a significant difference between HL and NHL groups in the distribution of viruses (p = 0.007) with higher frequency of the two viruses together in NHL (47%) and higher frequency of single virus infection in HL (15% of HHV6 and 18% of CMV, versus 6% for each in NHL). Lymphoma patients positive for both herpes viruses (n = 10) were 7 males and 3 females, with a median age of 6.5 years (range, 3-13 years).

**Table 3:** Distribution of herpes viruses as detected by PCR

	HL n = 33	NHL n = 17	p value
**HHV6, n(%)**	5(15)	1(6)	**0.007**
**CMV, n(%)**	6(18)	1(6)	
**Both, n(%)**	2(6)	8(47)	
**Negative cases, n(%)**	20(61)	7(21)	

**CMV Ab, median (range)**	2.9 (0-7.5)	3.0 (0-6.1)	0.902

NHL: Non Hodgkin's lymphoma, HL: Hodgkin's lymphoma, HHV6: herpes virus 6 single infection, CMV: cytomegalovirus single infection, Both: positive for HHV6/CMV DNA, Negative: negative for herpes viruses, CMVAb: cytomegalovirus IgG antibody titer

Concerning the presence of the each virus regardless of the other, CMV infection was significantly higher in NHL (9/17, 53%) compared to HL disease (8/33, 24%) (p = 0.042) and HHV6 DNA (in WBCs or plasma) was significantly higher in NHL (9/17, 53%) compared to HL disease (7/33, 21%) (p = 0.023).

In relation to lymphoma subtypes, in HL patients, we found that 2 (6%) patients of MC subtype were positive for HHV6 single infection and another 2 (6%) were positive for both HHV6/CMV. In NHL patients, we observed that eight (47%) patients of B-lineage subtype were positive for both herpes viruses (HHV6/CMV) (Table 2).

There was no significant difference in CMV IgG level between lymphoma and control groups [median 3 (0.0-7.5) vs. 2.4 (0.0-5.0), respectively, p = 0.109]. Also, CMV IgG level was not significantly different between HL and NHL patients (p = 0.902) (Table 3). However, CMV viremic lymphoma patients had significantly higher CMV IgG levels compared to those without viremia (median value was 4.0 (0.0-7.5) vs. 2.6 (0.0-5.7), (p = 0.006).

### The presence of herpes viruses (HHV6 and/or CMV) and clinico-pathological parameters in lymphoma patients

The detailed relation between HHV6 and/or CMV detection and clinico-pathological parameters is presented in table (IV). We compared patients negative for both viruses to those with positive HHV6 and/or CMV DNA. There was no significant difference between age groups (< 9 yrs and ≥ 9 yrs) in the presence of herpes viruses (p = 0.395). Similarly, there was no gender difference (p = 0.670) and no association between herpes virus infection and disease stage (p = 1.000). Presence of HHV6/CMV was significantly higher among patients under salvage therapy compared to those under induction and maintenance chemotherapy (p = 0.034). There was significantly higher frequency of HHV6/CMV in patients with uncontrolled disease (stationary or progressive) (p = 0.021). Two out of 50 lymphoma patients (4%) died, one of them was infected with HHV6 and the other was infected with CMV (Table 4).

**Table 4:** Relation between demographic and disease characteristics and distribution of herpes viruses among pediatric lymphoma patients

Factor	HHV6 n(%)	CMV n(%)	Both n(%)	Negative n(%)	p value
**Age**					
**< 9 y n = 25**	3(12)	3(12)	7(28)	12(48)	0.395
**≥9 Y n = 25**	3(12)	4(16)	3(12)	15(60)	
**Sex**					
**M: **n = 32	3(9)	4(13)	7(22)	18(56)	0.670
**F: **n = 18	3(17)	3(17)	3(17)	9(50)	
**Stage**					
I+II (n = 25)	3(12)	5(20)	4(16)	13(52)	1.000
III+IV (n = 25)	4(16)	3(12)	5(20)	13(52)	
**Phase of therapy**					
Induction: n = 24	2(8)	6(25)	5(21)	11(46)	
Maintenance: n = 23	3(13)	1(4)	3(13)	16(70)	**0.034**
Salvage: n = 3	1(33)	0(0)	2(67)	0(0)	
**Status of disease**					
CR+PR: n = 42	5(12)	6(14)	6(14)	25(60)	**0.021**
SD+PD: n = 8	2(25)	1(13)	4(50)	1 (13)	
**Outcome**					
**Af: **n = 38	4(11)	6(16)	6(16)	22(59)	
**Ad: **n = 10	1(10)	0(0)	4(40)	5(50)	*
**D: **n = 2	1(50)	1(50)	0(0)	0(0)	

CR: Complete remission, PR: Partial remission, SD: Stationary disease, PD: Prolonged disease, Af: Alive free of disease, Ad: Alive with disease, D: Died, HHV6: herpes virus 6 single infection, CMV: cytomegalovirus single infection, Both: positive for HHV6/CMV DNA, Negative: negative for herpes viruses

* No p value because only two patients died.

### The presence of herpes viruses (HHV6 and/or CMV) and some clinical parameters in lymphoma patients

Mucositis and other infections (cannula site infection, perianal infection, fungal chest infection, herpetic skin lesions, positive blood cultures for *Candida kruis*, *Klebsiella pneumonia*, mixed *Candida *species and *Aspergillus flavus*) were significantly associated with herpes virus infection (p < 0.001 and = 0.004, respectively). All patients with mucositis were positive for both herpes viruses DNA (70%), H6 alone (20%) or CMV alone (10%). On the other hand, 58% of those with other infections were positive for both HHV6/CMV DNA (Table 5).

**Table 5:** Herpes viruses and clinical parameters in pediatric lymphoma patients

Factor	HHV6 n(%)	CMV n(%)	Both n(%)	Negative n(%)	p value
**Fever **n = 22	3(14)	3(14)	7(32)	9(40)	0.100
**Rash **n = 9	0(0)	1(11)	2(22)	6(67)	0.479
**Organomegaly **n = 13	2(15)	1(8)	2(15)	8(62)	0.526
**Med. mass **n = 18	2(11)	4(22)	2(11)	10(56)	0.869
**Mucositis **n = 10	2(20)	1(10)	7(70)	0(0)	**< 0.001**
**Other infection **n = 14	3(21)	0(0)	8(58)	3(21)	**0.004**
**Duration of FN **(n = 28)					
Low Risk (< 10 d) n = 15	1(7)	3(20)	2(13)	9(60)	0.136
High Risk (≥ 10 d) n = 13	2(15)	0(0)	7(54)	4(31)	
**ANC (Median = 0.8)**					
Mild(≥ 0.8)n = 25	3(12)	6(24)	1(4)	15(60)	0.570
Severe (< 0.8)n = 25	3(12)	1(4)	9(36)	12(48)	
**Plt count**					
≥96 n = 25	4(16)	5(20)	2(8)	14(56)	1.000
< 96 n = 25	2(8)	2(8)	8(32)	13(52)	
**Hb**					
≥ 9.1 n = 25	3(12)	5(20)	1(4)	16(64)	0.256
< 9.1 n = 25	3(12)	2(8)	9(36)	11(44)	
**Mono**					
≥ 0.4 n = 25	3(12)	5(20)	2(8)	15(60)	0.570
< 0.4 n = 25	3(12)	2(8)	8(32)	12(48)	
**Lymph**					
≥ 0.5 n = 25	3(12)	4(16)	1(4)	17(68)	0.089
< 0.5 n = 25	3(12)	3(12)	9(36)	10(40)	

Med. Mass: Mediastinal mass, FN: Febrile neutropenia, ANC: absolute neutrophil count, Plt: Platelet count, Hb: hemoglobin concentration, Mono: monocyte, Lymph: Lymphocyte, HHV6: herpes virus 6 single infection, CMV: cytomegalovirus single infection, Both: positive for HHV6/CMV DNA, Negative: negative for herpes viruses

Presence of both herpes viruses was associated with more frequent episodes of febrile neutropenia (median 3 episodes vs. 0 episodes in negative herpes viruses cases) (p = 0.006). The presence of both herpes viruses was associated with high risk clinical parameters (long duration of febrile neutropenia (more than 10 days) (p = 0.080), ANC < 0.8×10^9^/l (p = 0.023), lymphopenia (< 0.5 ×10^9^/l) (p = 0.008), thrombocytopenia (Plt < 96 ×10^9^/l) (p = 0.137), and low Hb level (< 9.1 g/dl) (p = 0.010), more than patients negative for herpes viruses.

## Discussion

The presence of HHV6 DNA was detected in 16/50 (32%) of our lymphoma patients, 6/16 (37.5%) of these patients were viremic. Our results were close to those reported by Di Luca et al. who detected HHV6 DNA in 29% of their cases [24]. Sumiyoshi et al. [25] have reported presence of HHV6 in 50-68% in patients with different malignant lymphomas. In the current study, the frequency of HHV6 DNA in HD cases was 21%. This figure lies between the very higher frequencies reported by Valente et al. (73%) [26] and the low frequency (12%) reported by Torelli et al. [27]. None of our healthy volunteers showed HHV6 infection.

Discrepancies in the results could be attributed to several reasons: i) All these studies have investigated the presence of HHV6 in lymphoid tissues only which indicate that the virus could be activated at sites other than peripheral blood compartment [28], ii) The wide spectrum of sensitivity of the techniques used in different laboratories [26], the amount of DNA analyzed, and the presence of variants of HHV6 which did not hybridize well to the primers used could lead to false negative results, iii) most of our patients had absolute neutropenia which may affect the positivity of PCR assay due to the presence of a low number of infected cells.

In contrast with Tailor et al. [5], we observed that there was a significant difference in frequency of HHV6 infection between NHL and HL cases (p = 0.023). Such difference in frequency of HHV6 in both NHL and HL might be linked to different treatment regimen given according to type of lymphoma [5].

CMV is considered to be one of the most important opportunistic infections occuring in lymphoma patients as a result of impairment in cell mediated immunity [29]. CMV pneumonia (CMVp) is to be one of the most common clinical presentations of CMV disease and is associated with considerable morbidity and mortality in patients with hematological malignancies [30,31]; disease-specific mortality rate reaches up to 30% in lymphoma patients [32]. Moreover, CMV retinitis [33] encephalitis and oesophagitis have been observed among patients with HL and NHL diseases [34]. In contrast to Hingmire et al (34), who reported that CMV infections are less common in HL and NHL patients who did not undergo allogeneic BMT, we could detect CMV infection in 34% of our lymphoma patients. Furthermore, it is noteworthy that the majority of the patients in our NHL series derived from a B-cell lineage 16/17 (94%). More than half of our NHL patients of B cell subtype (9/16, 56%) showed CMV infection. This might be due to exposure to more selective suppressive chemotherapy that leads to diminished T cell function with the disappearance of CD8 cytotoxic population [29]. We also observed that the antibody titer to CMV was consistent with its reactivation from latency in lymphoma (median value in patients with and without CMV infection was 4, 2.6 respectively, p = 0.006).

It has been documented that the risk of developing severe CMV disease depends on the degree of immunosuppression and underlying disease [35]. Previous studies have reported that HHV6 infection is one of the major contributions for induction of an immunosuppression state in patients with BMT and solid organ transplantation. Multiple studies suggested association between HHV6 and CMV infection following organ transplantation with effects on both clinical picture and prognosis [25,36]. These observations encouraged us to investigate frequency of lymphoma patients positive for both herpes viruses (HHV6/CMV) and secondly, determine if there is a relationship between presence of both viruses and severity of lymphoma disease. Our data showed that 20% of lymphoma patients were positive for both herpes viruses. Moreover, the presence of both viruses was more common among NHL cases (8/17, 47%). Previous studies have addressed explanations for such observations which could be due to: (i) immunosuppression from both NHL disease and its treatment may predispose patients to higher risk of coinfection, (ii) An immunomodulating effect of HHV6 since it can induce production of interleukin-1β and tumor necrosis factor-alpha, suppress T lymphocyte function due to reduced interleukin-2 synthesis [7], and suppress bone marrow by inducing interferon-alpha production. [37,38], (iii) HHV-6 can directly infect CD4+ T-cells and induce apoptosis, thus altering key immune activation molecules pathways and subsequently disturbing the cytokine network. (iv) HHV-6 can also infect thymic epithelial cells, hematopoietic stem cells, and natural killer cells, which are critical for immune maturation and protection against cancer and viral infections.

All these factors could contribute to pathologic effects of other viral infections like CMV as a result of HHV6 reactivation [39], and also create an environment suitable for persistence of HHV6 latency [40].

The presence of both herpes viruses was associated with uncontrolled lymphoma disease (p = 0.021) and salvage phase of treatment (p = 0.034) but not with advanced stage (p = 1.000).

Griffiths et al. [41] showed that the combination of both HHV6 and CMV infection after organ transplantation was more likely to be associated with CMV disease than with CMV infection alone. Our observations extended to demonstrate the immunosuppressive effect of HHV6 and its associated clinical manifestations among cases who had CMV infection (reactivation or reinfection), these include: i) patients who were positive for CMV and negative for HHV6 were older than those with negative CMV and positive HHV6, median age were 15 y and 5.5 Y respectively. ii) 70% of patients with positive herpes viruses (HHV6, CMV) showed clinical manifestations of severe chest infection and were significantly associated with more frequent episodes of febrile neutropenia (median 3 episodes), long duration of febrile neutropenia > 10 days, absolute neutrophil count (ANC) of < 0.8, lymphopenia (< 0.5), and low Hb concentration (Hb < 9.1). iii) The presence of HHV6 single infection was significantly associated with longer duration of febrile neutropenia > 10 days when compared to HHV6 negative patients and did not show any significant difference when compared to those positive for both herpes viruses. However, we could not rely completely on such observations because all of our patients were subjected to lymphoma treatment which could aggravate the suppressive effect of HHV6, this favoring development of CMV infection and disease and because of the lower number of studied cases. Therefore, the role of HHV6 as a predictor for CMV syndrome in cases with CMV infection is needed to be carefully evaluated in a larger number of lymphoma cases. It will be also worthy to follow the patients before and after treatment to consider the role of iatrogenic immunosuppression.

In the present exploratory study, a single sample was analyzed. This could not indicate the timing, duration and the course of infection. More frequent sampling with quantitative analysis of peripheral blood and tissues in follow up studies may be necessary for better determining the causal effect of this unique β-herpesvirus relationship.

## In conclusion

the presence of HHV6 could be considered as a predicting indicator of cellular immunosuppression preceding the onset of CMV infection (reactivation or reinfection) which may result in a severe outcome among pediatric lymphoma patients. The presence of both herpes viruses appeared to be associated with an aggressive form of the lymphoma disease. The severe clinical manifestations associated with HHV6 single infection may suggest the synergistic effect of HHV6 on CMV associated infection.

## Competing interests

The authors declare that they have no competing interests.

## Authors' contributions

SA put plan and design of the study, carried out the practical part of the study and editing the manuscript. MF participated in the design of the study, carried out full clinical investigations to the studied children, evaluated the results and participated in editing clinical part of the study. ME have collected samples, collected clinical and hematological data from patient's sheets, drafted the data in tables, and participated in revising the important intellectual contents. MM has performed the statistical analysis and participated in editing and revising the manuscript.

All authors read and approved the final manuscript.
